# Docosahexaenoyl ethanolamide improves glucose uptake and alters endocannabinoid system gene expression in proliferating and differentiating C2C12 myoblasts

**DOI:** 10.3389/fphys.2014.00100

**Published:** 2014-03-21

**Authors:** Jeffrey Kim, Morgan E. Carlson, Bruce A. Watkins

**Affiliations:** Center on Aging, University of Connecticut Health CenterFarmington, CT, USA

**Keywords:** endocannabinoid system, C2C12 myoblasts, cannabinoid receptors, glucose uptake, gene expression, DHEA, polyunsaturated fatty acids

## Abstract

Skeletal muscle is a major storage site for glycogen and a focus for understanding insulin resistance and type-2-diabetes. New evidence indicates that overactivation of the peripheral endocannabinoid system (ECS) in skeletal muscle diminishes insulin sensitivity. Specific n-6 and n-3 polyunsaturated fatty acids (PUFA) are precursors for the biosynthesis of ligands that bind to and activate the cannabinoid receptors. The function of the ECS and action of PUFA in skeletal muscle glucose uptake was investigated in proliferating and differentiated C2C12 myoblasts treated with either 25 μM of arachidonate (AA) or docosahexaenoate (DHA), 25 μM of EC [anandamide (AEA), 2-arachidonoylglycerol (2-AG), docosahexaenoylethanolamide (DHEA)], 1 μM of CB1 antagonist NESS0327, and CB2 inverse agonist AM630. Compared to the BSA vehicle control cell cultures in both proliferating and differentiated myoblasts those treated with DHEA, the EC derived from the n-3 PUFA DHA, had higher 24 h glucose uptake, while AEA and 2-AG, the EC derived from the n-6 PUFA AA, had lower basal glucose uptake. Adenylyl cyclase mRNA was higher in myoblasts treated with DHA in both proliferating and differentiated states while those treated with AEA or 2-AG were lower compared to the control cell cultures. Western blot and qPCR analysis showed higher expression of the cannabinoid receptors in differentiated myoblasts treated with DHA while the opposite was observed with AA. These findings indicate a compensatory effect of DHA and DHEA compared to AA-derived ligands on the ECS and associated ECS gene expression and higher glucose uptake in myoblasts.

## Introduction

Skeletal muscle serves as a major target organ for glucose removal from circulation and the relevancy of this tissue is bolstered by the disease states of insulin resistance and diabetes. Under euglycemic conditions in healthy subjects, approximately 75% of glucose removal is mediated by non-insulin stimulated glucose uptake primarily by the brain and to a lesser extent in other tissues such as skeletal muscle (Baron et al., 1988). However, under hyperglycemic conditions, glucose uptake mediated via non-insulin stimulated glucose uptake increases considerably (Capaldo et al., 1986). Moreover, the importance of basal glucose uptake is apparent during insulin resistance and diabetes when close to 80% of glucose uptake is achieved postprandially (Best et al., 1996).

In recent years, researchers have identified a physiologic mechanism that regulates the balance of macronutrient metabolism. The endocannabinoid system (ECS) is a complex network encompassing various physiological systems in the body that is comprised of the G-protein-coupled receptors, CB1 and CB2, their lipid-derived endogenous ligands termed endocannabinoids, and the enzymes that are involved in the biosynthesis/degradation of the endocannabinoids. While the ECS has been shown to influence several physiological activities, such as hunger, pain modulation, mood, and inflammation, the primary function appears to impact energy homeostasis, as activation of the ECS appears to shift energy balance toward energy storage (De Petrocellis et al., 1999; Soderstrom et al., 2004; Valenti et al., 2005; Piazza et al., 2007).

Previous findings confirm the role of the ECS in food intake and energy homeostasis. Reduced food intake was reported in mice (Despres et al., 2005) by pharmacologically antagonizing CB1 as well as in CB1 knockout mice (Pi-Sunyer et al., 2006). The interest in targeting this specific cannabinoid receptor is supported by several key findings. Importantly, antagonism of CB1 reduced food intake and body weight (Cota et al., 2003; Ravinet Trillou et al., 2003). However, the reduced food intake did not account for the total reduction in body weight, suggesting an ulterior means of energy expenditure. Furthermore, in both obese subjects and in leptin deficient mice, reduced glucose uptake and fatty acid oxidation were observed to be reversed by antagonizing CB1 (Liu et al., 2005; Cavuoto et al., 2007). Along with this, blood concentrations of the two chiefly studied endocannabinoids, anandamide (AEA) and 2-arachidonoylglycerol (2-AG) both derived from arachidonic acid (AA), are elevated in obese compared to lean individuals (Bluher et al., 2006; Matias et al., 2006).

In support of CB1 exerting a role on energy balance, evidence from several studies in animal models as well as in humans revealed that in conditions of obesity and hyperglycemia the ECS is in an overactivated state (Engeli et al., 2005; Bluher et al., 2006; Matias et al., 2006). This phenomenon is referred to as ECS tone, which is the collective actions of ligands, receptors, and enzymes of endocannabinoid synthesis and degradation. In other words, the ECS is overactivated in the state of obesity. Thus in the overactivated state a dysregulated ECS observed in both the obese and an insulin resistive state contributes to greater lipid accumulation and insulin resistance in muscle. Targeting the ECS to improve signaling for maintaining healthy muscle glucose sensitivity and reduce excessive lipid accumulation is an attractive application. Moreover findings that the ECS plays an active role in macronutrient metabolism we propose that directing endogenous agonist actions on the ECS to improve glucose homeostasis has implications for diabetes and obesity.

Recognizing that dietary sources of fatty acids and more specifically the polyunsaturated fatty acids (PUFA) can alter tissue composition of membrane phospholipids, dietary manipulation permits indirect control of the ECS on cellular functions. It is well known that the amount and type of dietary n-6 and n-3 PUFA changes the PUFA composition of membrane phospholipids in various tissues including muscle and bone compartments (Brown et al., 1991; Watkins et al., 2003; Hutchins-Wiese et al., 2012). Additionally, studies on mouse adipocytes demonstrated that cultures incubated with AA or docosahexaenoic acid (DHA) for 72 h elevated levels of these PUFA in phospholipids compared to the vehicle controls (Matias et al., 2008). Furthermore, AA treatment in the adipocyte cultures led to an increase of 2-AG concentrations while DHA treatment significantly decreased both AEA and 2-AG concentrations compared to the vehicle control.

Various studies on feeding rodents different dietary PUFA resulted in alterations in the levels of endocannabinoids of brain (Watanabe et al., 2003), adipose (Batetta et al., 2009), and liver (Artmann et al., 2008; Batetta et al., 2009). Mice fed a high fat diet supplemented with krill oil for 8 weeks were found to have reduced levels of AA-derived endocannabinoids in various peripheral tissue, including gastrocnemius (Piscitelli et al., 2011). Changes were reported in ECS genes of muscle when different dietary PUFA were fed to mice. For example, in our laboratory, analysis of quadriceps muscle from male ND4 Swiss Webster mice fed either a control diet (AIN-93G with safflower oil) or a high n-3 PUFA diet (EPA+DHA 17.6 g/kg) for 26 days revealed changes in several ECS genes (Hutchins-Wiese et al., 2012). We reported that the mRNA for AEA synthesis enzyme, N-acyl phosphatidylethanolamine phospholipase (NAPE-PLD), and 2-AG synthesis enzyme, diacylglycerol lipase (DAGL)α and DAGLβ, were higher in muscle of mice fed the n-3 PUFA diet compared to the controls. Furthermore, in this study the mRNA expression of CB1 and CB2 were both higher in muscle of mice fed the high n-3 PUFA diet. Collectively, these findings strongly suggest that dietary n-3 PUFA treatment can alter mRNA expression of key ECS genes to influence ECS signaling.

Since cellular membranes participate in numerous physiological and biochemical processes, changing the PUFA composition of cell membranes through diet can alter signaling and receptor functions associated with the ECS. Based on these findings that ECS gene expression changed upon exposure to different amounts and families of PUFA, the ECS is likely to adapt and change accordingly to these nutrients that serve as EC precursors. Therefore, an investigation exploring the adaptations from dietary PUFA enrichment on the skeletal muscle ECS is warranted as this signaling system is actively involved in energy homeostasis.

To investigate the role of the ECS on basal glucose uptake, our overall research hypothesis was that dietary long chain n-3 PUFA, DHA, and EPA, enrichment of C2C12 myoblasts restores endocannabinoid tone (action of ligands, receptors, and enzymes of the ECS synthesis and degradation) and signaling of this system to improve glucose homeostasis with potential applications to reduce obesity and diabetes. Since ECS receptor activation likely plays a role in impacting glucose uptake, the investigation also evaluated the consequences of PUFA treatment with cannabinoid receptor agonists and antagonists. The specific aim of this research was to describe the effects of PUFA enrichment on endocannabinoid gene expression and agonist/antagonist actions on glucose homeostasis in the myoblast cell line C2C12. Herein, C2C12 myoblast cell cultures were enriched with 25 μM levels of PUFA and the mRNA (qPCR) and protein expression (Western blot) of endocannabinoid enzymes and cannabinoid receptors were quantified. The expression of these enzymes and receptors were studied in myoblast cell cultures in both the proliferative and differentiation committed states. Additionally, insulin signaling/glucose uptake was measured as a consequent outcome.

## Materials and methods

### Cell cultures and treatments

C2C12 cells were purchased from American Type Culture Collection (ATCC, Manassas, VA, USA) and routinely cultured in growth media (GM) consisting of Dulbecco's Modified Eagles Medium (DMEM; Thermo Fisher Scientific, Waltham, MA, USA) supplemented in 10% fetal bovine serum (FBS, Thermo Fisher Scientific, Waltham, MA, USA), and 1% antibiotic solution or in differentiation media (DM), consisting of DMEM with 2% horse serum (Thermo Fisher Scientific, Waltham, MA, USA) in place of FBS. Cultures were maintained in vented 75 cm^2^ tissue culture flasks (Becton Dickinson and Company, Franklin Lakes, NJ, USA) at a density of 1 × 10^6^ cells per flask as determined by automated TC-10 cell counter (Bio-Rad laboratories, Inc., Hercules, CA, USA). Seeded at this density, cultures became 80–85% confluent after 72 h, at which time the cells were passed onto new flasks containing fresh GM. C2C12 cell cultures in all experiments were from passages 5–9.

Proliferating and differentiated cells were used to model skeletal muscle, such as within an *ex vivo* model or whole organism. Skeletal muscle found in mammals contains a mix of both proliferating and differentiating myoblasts (during active regeneration from injury, in addition to routine organ maintenance and homeostasis). Differentiated C2C12 were chosen to mimic mature myofibers. Myogenin and MyoD1, markers of differentiation, were used to verify that myoblasts had committed toward differentiation.

### Chemicals and reagents

The treatment media contained PUFA AA, EPA, and DHA all from Nu-Chek-Prep, Inc. (Elysian, MN, USA) and endocannabinoids (AEA and 2-AG from Abcam, PLC., Cambridge, MA, USA) that were dissolved in 100% ethanol at a final concentration of 100 mg/mL, flushed with N_2_ and stored in glass amber vials at −20°C until needed. The PUFA containing media were prepared by adding fatty acid stock aliquots to either serum free GM containing endotoxin/fatty acid free BSA (Sigma Chemical Company, Saint Louis, MO, USA) that was used at a concentration dependent of PUFA concentration (2:1, PUFA:BSA). Working concentrations of the PUFA stock solutions were diluted as appropriate to achieve the necessary final concentrations. Cell cultures were treated for 24 h in 37°C at 5% CO_2_. 24 h prior to cell collection then treated with varying physiologic concentrations of AA, EPA, DHA, AEA, or 2-AG at 25 μM while 5, 10, and 25 μM for the glucose uptake experiments. Additionally, NESS0327, a CB1 antagonist, or AM630, a CB2 inverse agonist were used to pretreat cells at concentrations of 1, 2, or 5 μM.

### Fatty acid methyl esters analysis of C2C12 cell cultures

Fatty acid methyl esters (FAME) analysis was performed to measure fatty acid composition in C2C12 myoblast cultures, which were washed with calcium/magnesium-free phosphate buffered saline (PBS; 137 mM NaCl, 2.7 mM KCl, 9.9 mM Na_2_HPO_4_, 1.8 mM KH_2_PO_4_; Thermo Fisher Scientific, Waltham, MA, USA) and removed by scraping with a Teflon scraper. Cells were sonicated and extracted for lipids with chloroform/methanol (2:1, vol/vol) (Thermo Fisher Scientific, Waltham, MA, USA). Extracted lipids were treated with 0.5 N NaOH in methanol, and FAME prepared by esterification using boron trifluoride (BF3) in methanol (10% w/w, Supelco Inc., Bellefonte, PA, USA). The FAME were concentrated in isooctane (HPLC grade, Fisher Scientific, Pittsburg, PA, USA) and analyzed by gas chromatography (GC) (HP 7890A series, autosampler 7693, GC ChemStation Rev.B.04.03, Agilent Technologies, Palo Alto, CA, USA) with a DB-225 column (30 m, 0.25 mm i.d., 0.15 mm film thickness, Agilent Technologies, Palo Alto, CA, USA) equipped with a flame ionization detector (Li et al., 2010). Sample peaks were identified by comparison to authentic FAME standards (Nu-Chek-Prep Inc., Elysian, MN, USA). Sample injection volume was 3 μL and split ratio 10:1. Results for FAME analysis were obtained by weight percentage reports based on the response values for authentic standards of known concentrations to determine weight percentages values. This approach facilitates lipid and subsequent FAME recovery to minimize losses in peak responses at lower concentrations of components.

### Quantitative real-time polymerase chain reaction (qPCR)

Analysis of mRNA expression of genes of interest was measured to understand changes in ECS and glucose-related genes after PUFA or endocannabinoid treatment. C2C12 cells were cultured in 75 cm^2^ flasks until 85–90% confluent, followed by treatment with fatty acid or endocannabinoid. Afterwards, cells were washed twice with cold PBS, followed by RNA extraction with TRIzol (Invitrogen Corp., Carlsbad, CA, USA) reagent. RNA samples were then treated with DNase I (Ambion, Carlsbad, CA, USA) to remove any DNA contamination. Total RNA (1 μg) was reverse transcribed to cDNA in a reaction mixture using RNA transcriptase superscript III (Invitrogen Corp., Carlsbad, CA, USA). Briefly, 9 μL of the following were combined for each sample: 4 μL 5X VILO reaction mix containing random hexamers and dNTPs, 2 μL 10X Superscript enzyme mix® (BioRad Laboratories, Hercules, CA, USA) containing RNA inhibitors, and 3 μL of DEPC treated water for a total volume of 20 μL. Samples were heated as specified for superscript RT: 25°C for 10 min, 42°C for 60 min, and 85°C for 5 min. Synthesized cDNA product was then used for quantitative RT-PCR. A master mix for RT-PCR was prepared with SsoFast EvaGreen® Supermix (BioRad Laboratories, Hercules, CA, USA). Briefly, 10 μL SsoFast EvaGreen® was mixed with 6 μL DEPC treated water, 1 μL of 10 μM forward primer, and 1 μL of 10 μM reverse primer. A total of 18 μL of master mix was added to wells of unskirted 96-well plates with 2 μL of cDNA. All samples were analyzed in triplicate. Fluorescence emission was detected and cycle threshold (CT) values were calculated in the linear range automatically. Relative CT amounts were calculated from the standard curve for each gene, which were normalized to the reference gene, GAPDH, afterwards. The primers sequences used in this work are shown in Table 1. ΔΔCT values for each sample were determined by calculating the difference between the CT value of the target gene and the CT value of the reference gene, GAPDH. The normalized level of expression of the target gene in each sample was calculated using the formula 2^−ΔΔCT^. Values were expressed as fold of the control.

**Table 1:** **Primer sequences used for quantitative polymerase chain reaction (qPCR)**.

**Gene**	**Forward**	**Reverse**	**Accession**
GAPDH	TGTGATGGGTGTGAACCACGAGAA	GAGCCCTTCCACAATGCCAAAGTT	GU214026.1
CB1	TCACACCTCAGAAGATGGCAAGGT	AGCAGATGATCAACACCACCAGGA	AY522554.1
CB2	TGAAGATCGGCAGTGTGACCATGA	AATGCTGAGAGGACCCACATGACA	U21681.1
NAPE-PLD	TGGCATTGTGCATGAAAGCCCTAC	AGTGGGCATGGTGTAGTTGTCAGT	AB112350.1
FAAH	TGGCATTGTGCATGAAAGCCCTAC	AGTGGGCATGGTGTAGTTGTCAGT	NM_010173.4
DAGL-α	CGACCACCAAGTGCAACCATTGAA	AACTCGGCGAATTCTAGCACCTGA	BC148308.2
DAGL-β	TGTGTGTCAGCATGAGAGGAACCA	GTGGCGATGATGCCAATGACAACT	BC016105.1
Akt-1	GTAGCCATTGTGAAGGAG	TCTTGAGGAGGAAGTAGC	AF124142.1
Insulin-R	TCCTGGAAATCGTCAACCTGCTCA	ACGATCCAACGGGACATTCTCCAT	J05149.1
IRS-1	GGCACATCTCCTACCATT	CATCATCTCTGTATATTCCTCAAT	NM_010570.4
GLUT4	TCGTGGCCATATTTGGCTTTGTGG	TAAGGACCCATAGCATCCGCAACA	AB008453.1
GLUT1	CATCGCCCTGGCCCTGCAGGAGC	GGCACCCCCCTGCCGGAAGCCGGA	D10229.1
Adiponectin	AATGACAGGAGCTGAAGGGC	AGGTGAAGAGAACGGCCTTG	NM_009605.4
Myogenin	CGTGGGCATGTAAGGTGTGTAAGA	CATTCACTTTCTTGAGCCTGCGCT	M95800.1
MyoD1	TGAGCAAAGTGAATGAGGCCTTCG	AGAGCCTGCAGACCTTCGATGTA	M18779.1
IL-6	ATCCAGTTGCCTTCTTGGGACTGA	TAAGCCTCCGACTTGTGAAGTGGT	M20572.1
TNF-α	AGCCGATGGGTTGTACCTTGTCTA	TGAGATAGCAAATCGGCTGACGGT	D84199.2
MCP-1	TGAGCCATGGGAACAAGGAAGTCT	TGTGCTGGTCTGTGATAGGCACAT	BC055070.1
AMPK-α2	TCCTGAAGACCCCTCCTACG	GAGTGGTTCTCAGCTGTGCT	NM_178143.2
Adenylyl cyclase	GAAAGTGCGAACCCAGAGGA	ACTTGCGGACGTGTTCAGAT	AF458089.1
p42/p44 (MAPK)	AGCCACACGTTGGTACAGAG	CCAGAGCTTTGGAGTCAGCA	X58712.1
p38 (MAPK)	GGCTGATGAGGAGATGACCG	ATGGGAGGCAGAGACTGGAT	AF135185.1
JNK (MAPK)	AGTGGGTTGCATCATGGGAG	CTGCATCTGAAGGCTGGTCT	NM_016700.4

### Western blot analysis

Protein expressions of genes were analyzed by collecting whole cell lysates after incubation with PUFA treatments. Cells were washed twice with cold PBS followed by scraping to dislodge from surface and transferred to 1.5 mL microfuge tubes to be lysated using a lysis buffer (100 mM Tris-HCl + 0.1% Triton X-100, pH 7.5). Lysates were then centrifuged at 12,000 g for 5 min at 4°C, after in which the supernatant was collected and stored at −80°C. Proteins were then separated by polyacrylamide gel (gradient 4–15%) electrophoresis then transferred to a PVDF membrane, to be followed by antibody incubation against the protein of interest (α-mouse-CB1, α-mouse-CB2, α-rabbit-GLUT4, α-rabbit-Insulin-R, Abcam, PLC., Cambridge, MA, USA). Protein expression was detected using Westpico horseradish peroxidase chemiluminescence and imaged using a Chemidoc XRS+ system and Image Lab software (BioRad Laboratories, Hercules, CA, USA) (Tsang et al., 1989).

### Flow cytometry

In addition to Western blot analysis, FACS flow cytometry was also used to measure expression of cannabinoid receptors. C2C12 cultures were washed twice with cold PBS followed by scraping to dislodge from surface and transferred to 1.5 mL microfuge tubes to be incubated with primary antibodies (α-goat-CB1 and α-rabbit-CB2, Abcam, PLC., Cambridge, MA, USA) diluted in FACS buffer (1:250) for 1 h in 4°C. Afterwards, cells were centrifuged at 500 g for 5 min (4°C) and washed 3 times. Following washing, secondary antibodies (goat α-rabbit 488 and donkey α-goat FITC; Thermo Scientific, Abcam, PLC, Cambridge, MA, USA) were diluted in FACS buffer (1:500) for 1 h in 4°C in the dark. Cells were then centrifuged at 500 g for 5 min (4°C) and washed 3 times. Cells were resuspended in 1 mL of FACS buffer for FACS flow cytometry. Cell acquisition was performed on a FACSCalibur flow cytometer (BD Biosciences, San Jose, CA). 10,000 events were processed for each measurement. Data was analyzed using FlowJo software (Tree Star Inc., Ashland, OR).

### Glucose uptake assay

Glucose uptake assay kits (Caymen Chem, Ann Harbor, MI) were used to assess and quantify glucose uptake capacity after fatty acid, endocannabinoid, or ECS receptor inhibitor treatment. Glucose is taken up by the majority of cells via the action of glucose transporters, which facilitate glucose movement down a concentration gradient, in contrast to energy-dependent uptake of glucose in the gut or kidney. Use of a recently developed fluorescent glucose analog, 2-deoxy-2-[(7-nitro-2,1,3-benzoxadiazol-4-yl) amino]-D-glucose) (2-NBDG), allows for the target investigation of glucose uptake without having to account for glucose metabolism or conversion to glycogen once taken up by cells. 2-NBDG is a fluorescently-labeled deoxyglucose analog and is used as the sole probe for detection.

Proliferating and differentiating C2C12 cells were seeded at ~5 × 10^4^ cells/well in black walled/clear bottom 96-well plates (Corning, Inc., Corning, NY, USA) in DMEM (+1% Pen/Strep and 10% FBS). Upon reaching a confluency of 50%, differentiation was induced with differentiating media consisting of high glucose DMEM, 2% Horse Serum (HS), and 1% Pen/Strep. After 48 h, media was changed to specific PUFA or EC containing media at a concentration of 5, 10, or 25 μM or cannabinoid receptor inhibitor (1, 2, or 5 μM) for 24 h. Following PUFA enrichment, media was removed from wells and treated with 100 μg/mL 2-NBDG for 1 h. Afterwards plates were centrifuged for 5 min at 500 g at room temperature and washed twice with the provided cell-based assay. 2-NBDG taken up by the cells was then measured at a wavelength of 485/535 nm (excitation/emission) on a BioTek Synergy HT plate reader (BioTek Instruments, Inc., Winooski, VT, USA).

### Statistical analyses

The results from experiments on gene expression and glucose uptake in proliferating and differentiated C2C12 cell cultures were analyzed for significance by One-Way ANOVA at *P* < 0.05. Where significant differences were found, a Tukey's Multiple Comparison Test was performed at a probability of α = 0.05 (SAS for Windows version 9.3, SAS Institute Inc., Cary, NC). The data are presented as means ± s.e.m. as well as means ± *SD* where indicated with *n* = 3 for each treatment group. These data were also expressed as standardized differences calculated from the difference between values of treatment and control divided by the mean of the control replicates [(treatment—BSA)/BSA * 100, BSA is the mean of 3 BSAs]. The results from experiments on CB1 and CB2 receptor expression in proliferating and differentiated C2C12 cell cultures were performed via FACS flow cytometry were analyzed for significance by One-Way ANOVA at *P* < 0.05. Where significant differences were found, a Tukey's Multiple Comparison Test was performed at a probability of α = 0.05. These data were also expressed as standardized differences calculated from the difference between values of treatment and control divided by the mean of the control replicates [(treatment—BSA)/BSA * 100, BSA is the mean of 3 BSA values]. Flow cytometry data were also compared among three concentrations for the same treatment by One-Way ANOVA. A Two-Way ANOVA analysis was further performed on flow cytometry data to detect any interactions between the various treatments and the three tested concentrations of each treatment. Results from the analysis of fatty acid methyl esters (FAME) by gas chromatography to reflect the fatty acid composition of enriched C2C12 cell cultures (proliferating and differentiated) were analyzed by a One-Way ANOVA at *P* < 0.05. Where significant differences were found, a Tukey's Multiple Comparison Test was performed at a probability of α = 0.05. The FAME data are expressed as means ± s.e.m. Each treatment group was comprised of three replicates.

Results from experiments on glucose uptake assay were performed in proliferating and differentiated C2C12 after PUFA or EC and inhibitor treatments (expressed as relative units of fluorescence). The collected data were analyzed for significance by one-way ANOVA at *P* < 0.05. Where significant differences were found, a Tukey's Multiple Comparison Test was performed at a probability of α = 0.05. These data are presented as means ± s.e.m. as well as means ± *SD* with *n* = 3 for each treatment group. Glucose uptake data were also adjusted by cell numbers expressed as relative units of fluorescence/5000 cells and same statistical procedures were applied to this set of data. Glucose uptake data were further expressed as standardized differences calculated from the difference between values of treatment and control divided by the mean of the control replicates [(treatment—BSA)/BSA * 100, BSA is the mean of 3 BSAs]. The same statistical procedure was applied to the adjusted and standardized data for the evaluation of results.

## Results

### Fatty acid analysis of lipids from C2C12 cell cultures

C2C12 myoblasts were cultured for 24 h following subculturing to allow cells to attach to flasks. Following 24 h, cells designated for differentiation were exposed to DM for 48 h, when myotubes formation was present and levels of myogenin and MyoD1 mRNA were elevated verified by RT-PCR. Both differentiated and proliferating cells were treated with either 25 μM of AA, EPA, DHA, AEA, 2-AG, or the no-fatty acid vehicle control, BSA. The fatty acid composition of total lipids extracted from differentiated C2C12 cells after enrichment is shown in Table 2 and for proliferating cells supplemental Table S1. The PUFA enrichment of cell cultures demonstrated a multiple fold increase in the respective PUFA enrichment and n-3 PUFA decreased the levels of AA. Likewise AA treatment resulted in lower levels of EPA and DHA in myoblast cultures. Treatment of cultures with AEA resulted in lower 18:2n6 but higher 20:1n9 in differentiated cells compared to the BSA vehicle control cell cultures. 2-AG treatment of differentiated myoblast cultures resulted in lower t16:1, 16:1n7, 18:1n9, 18:1n7, 18:2n6, and 20:5n3 and higher 20:1n9, 20:4n6, 22:4n6, and 22:5n3 (Table 2). Proliferating C2C12 cell cultures treated with 2-AG had lower t16:1, 16:1n7, 18:1n9, 18:1n7, and 18:2n6 and higher 20:4n6, 22:4n6 (Table S1).

**Table 2:** **Fatty acid composition of total lipids from differentiated C2C12 cell cultures**.

**Fatty acid**	**Treatments**						**Pooled s.e.m**	**ANOVA *p* value**
	**BSA**	**AA**	**EPA**	**DHA**	**AEA**	**2-AG**		
14:0	1.07^a^	0.68^b^	0.65^b^	0.71^b^	1.02^a^	1.05^a^	0.05	<0.0001
14:1n5								
15:0								
16:0	13.70^a^	11.39^c^	11.97^b^	12.21^b^	13.42^a^	13.32^a^	0.10	<0.0001
t16:1	2.31^a^	1.42^d^	1.49^d^	1.74^c^	2.29^a^	2.09^b^	0.03	<0.0001
16:1n7	4.64^a^	2.49^d^	2.70^cd^	2.88^c^	4.65^a^	3.81^b^	0.05	<0.0001
17:0	0^b^	0^b^	0.37^a^	0.18^ab^	0.08^b^	0.07^b^	0.06	0.0066
18:0	13.74^ab^	12.44^b^	13.56^ab^	13.05^ab^	14.15^a^	14.05^a^	0.31	0.018
18:1n9	19.80^a^	11.67^e^	12.90^d^	13.95^c^	19.69^a^	16.50^b^	0.15	<0.0001
18:1n7	7.04^a^	4.91^d^	5.06^cd^	5.27^c^	7.12^a^	6.32^b^	0.05	<0.0001
18:2n6	4.28^a^	2.11^e^	3.49^d^	3.70^c^	4.01^b^	3.52^d^	0.03	<0.0001
18:3n6	0.11^b^	0.70^a^	0.29^b^	0^b^	0.19^b^	0.16^b^	0.07	0.0002
18:3n3								
20:0								
20:1n9	0^b^	0^b^	0^b^	0^b^	0.32^a^	0.19^a^	0.04	0.0002
20:2n6								
20:3n6	1.79^a^	0.28^c^	1.04^b^	1.34^ab^	1.68^a^	1.39^ab^	0.12	<0.0001
20:4n6	9.31^c^	33.94^a^	6.37^e^	7.06^d^	9.20^c^	15.58^b^	0.18	<0.0001
20:3n3								
20:5n3	1.87^c^	0.69^e^	21.57^a^	4.41^b^	1.75^c^	1.66^d^	0.22	<0.0001
22:0	0	0	0.08	0	0	0	0.03	0.46
22:1n9								
22:4n6	0.83^c^	4.09^a^	0.67^d^	0.48^e^	0.84^c^	1.22^b^	0.01	<0.0001
22:5n6								
22:5n3	3.17^c^	2.45^d^	7.03^a^	1.94^e^	3.18^c^	3.40^b^	0.04	<0.0001
22:6n3	3.68^b^	2.23^c^	2.29^c^	22.09^a^	3.84^b^	3.73^b^	0.06	<0.0001
24:0	0.10	0	0	0	0.08	0.17	0.06	0.35
24:1n9	0.22	0	0.10	0.13	0.20	0.21	0.10	0.61
Saturated	28.61^ab^	24.51^d^	26.62^bc^	26.15^cd^	28.75^a^	28.67^ab^	0.43	<0.0001
Monounsaturated	31.70^a^	19.08^e^	20.76^d^	22.23^c^	31.98^a^	27.03^b^	0.18	<0.0001
Total PUFA	25.04^d^	46.49^a^	42.76^b^	41.01^b^	24.69^d^	30.65^c^	0.42	<0.0001
n-6 PUFA	16.32^c^	41.11^a^	11.87^d^	12.58^d^	15.92^c^	21.87^b^	0.19	<0.0001
n-3 PUFA	8.72^c^	5.37^d^	30.89^a^	28.44^b^	8.77^c^	8.79^c^	0.28	<0.0001
Ratio of n-6/n-3	1.87^c^	7.65^a^	0.38^d^	0.44^d^	1.81^c^	2.49^b^	0.01	<0.0001

Data represent means of n = 3 for each group and the pooled s.e.m. Values within rows having different superscripts are significantly different by One-way ANOVA and Tukey's mean separation test at α = *0.05*. BSA, bovine serum albumin; AA, arachidonic acid; EPA, eicosapentaenoic acid; DHA, docosahexaenoic acid; AEA, anandamide; 2AG, 2-arachidonoyl glycerol

### DHA treatment leads to a compensatory expression of ECS-related genes suggesting a dampening of ECS activation

Analysis of mRNA by quantitative RT-PCR from proliferating and differentiated C2C12 myoblasts that were treated with 25 μM of either AA, EPA, DHA, AEA, 2-AG or a no-fatty acid BSA vehicle control revealed several changes on ECS- and glucose uptake -related mRNA expression when normalized to the housekeeping gene GAPDH. These data were pooled for analysis from two separate experiments (Table 3 differentiated cell cultures and Table S2 proliferating cell cultures).

**Table 3:** **mRNA expression in differentiated C2C12 myoblast cultures treated with PUFA and EC**.

**Measurement**	**BSA**	**AA**	**DHA**	**EPA**	**AEA**	**2-AG**	**EPEA**	**DHEA**	**Pooled s.e.m.**	**ANOVA *p* value**
CB1	1.0^e^	0.7^f^	2.1^b^	1.5^d^	0.8^f^	0.8^f^	1.8^c^	2.3^a^	0.02	<0.0001
CB2	1.0^d^	0.6^e^	2.3^a^	1.5^c^	0.7^e^	0.8^de^	1.8^b^	0.8^de^	0.1	<0.0001
NAPE-PLD	1.0^c^	0.7^e^	0.8^de^	1.6^a^	0.5^f^	0.9^cd^	1.7^a^	1.2^b^	0.03	<0.0001
FAAH	1.0^d^	1.5^c^	0.9^d^	2.8^a^	2.4^b^	1.1^d^	1.6^c^	1.5^c^	0.1	<0.0001
DAGL-α	1.0^c^	2.4^b^	0.9^cd^	3.4^a^	1.0^c^	0.7^d^	1.1^c^	1.0^c^	0.0	<0.0001
DAGL-β	1.0^cd^	2.1^b^	0.8^de^	3.4^a^	1.1^c^	0.6^e^	1.1^c^	1.1^cd^	0.1	<0.0001
Akt-1	1.0^de^	0.9^ef^	1.7^b^	1.3^c^	0.8^f^	1.0^d^	1.0^de^	2.6^a^	0.03	<0.0001
Insulin r	1.0^c^	0.8^d^	1.7^b^	1.1^c^	0.7^d^	0.8^d^	1.1^c^	2.4^a^	0.04	<0.0001
IRS-1	1.0^c^	0.8^d^	1.5^b^	1.1^c^	0.1^e^	0.1^e^	1.1^c^	1.7^a^	0.02	<0.0001
GLUT4	1.0^bc^	0.9^cd^	3.1^a^	1.0^bc^	0.8^d^	0.8^d^	1.1^b^	3.3^a^	0.05	<0.0001
GLUT1	1.0^c^	0.7^d^	1.5^b^	1.1^c^	0.2^e^	0.3^e^	1.1^c^	3.8^a^	0.02	<0.0001
Myogenin	1.0^b^	1.2^b^	1.6^a^	1.1^b^	1.1^b^	1.1^b^	1.1^b^	1.0^b^	0.04	<0.0001
MyoD1	1.0^b^	1.2^b^	1.5^a^	1.2^b^	1.2^b^	1.1^b^	1.0^b^	1.0^b^	0.04	<0.0001
IL-6	1.0^c^	1.5^b^	0.5^d^	1.1^c^	1.9^a^	1.6^b^	0.6^d^	0.5^d^	0.02	<0.0001
TNF-α	1.0^a^	0.7^bc^	0.7^b^	1.0^a^	0.6^cd^	0.5^cd^	1.0^a^	0.5^d^	0.03	<0.0001
AMPK α2	1.0^d^	1.0^d^	1.6^b^	1.1^d^	0.7^e^	0.7^e^	1.3^c^	1.8^a^	0.02	<0.0001
Adenylyl Cyclase	1.0^a^	1.0^a^	1.0^a^	1.0^a^	0.3^d^	0.2^d^	0.8^b^	0.6^c^	0.02	<0.0001
p42/p44 (MAPK)	1.0^e^	1.4^d^	0.8^f^	0.7^f^	1.7^c^	1.6^c^	1.9^b^	2.9^a^	0.03	<0.0001
p38 (MAPK)	1.0^cd^	1.2^bcd^	0.8^d^	0.9^d^	1.5b^c^	1.3^bcd^	1.6^b^	3.8^a^	0.12	<0.0001
JNK (MAPK)	1.0^e^	1.1^d^	0.8^f^	0.9^ef^	1.4^c^	1.3^cd^	1.9^b^	3.5^a^	0.03	<0.0001
**STANDARD DEVIATION**										
CB1	0.01	0.1	0.00001	0.04	0.01	0.1	0.02	0.1		
CB2	0.03	0.01	0.2	0.2	0.02	0.02	0.02	0.02		
NAPE-PLD	0.03	0.03	0.03	0.04	0.05	0.1	0.1	0.03		
FAAH	0.03	0.1	0.1	0.1	0.2	0.04	0.02	0.1		
DAGL-α	0.04	0.1	0.1	0.2	0.1	0.05	0.1	0.03		
DAGL-β	0.1	0.1	0.1	0.2	0.04	0.04	0.1	0.1		
Akt-1	0.1	0.001	0.02	0.1	0.04	0.04	0.04	0.1		
Insulin r	0.1	0.03	0.1	0.1	0.02	0.03	0.03	0.1		
GLUT4	0.05	0.1	0.04	0.2	0.04	0.03	0.01	0.1		
Myogenin	0.1	0.04	0.1	0.1	0.002	0.1	0.001	0.03		
MyoD1	0.04	0.1	0.1	0.1	0.1	0.05	0.05	0.03		
GLUT1	0.02	0.01	0.02	0.1	0.01	0.01	0.1	0.1		
IRS-1	0.02	0.01	0.04	0.1	0.001	0.001	0.03	0.05		
IL-6	0.03	0.1	0.01	0.1	0.02	0.1	0.01	0.01		
TNF-α	0.1	0.01	0.03	0.1	0.01	0.02	0.04	0.01		
AMPK α2	0.02	0.02	0.05	0.1	0.1	0.03	0.03	0.03		
Adenylyl Cyclase	0.02	0.03	0.1	0.1	0.01	0.01	0.04	0.02		
p42/p44 (MAPK)	0.04	0.04	0.03	0.03	0.02	0.1	0.1	0.1		
p38 (MAPK)	0.02	0.02	0.02	0.1	0.0005	0.05	0.4	0.4		
JNK (MAPK)	0.01	0.04	0.02	0.1	0.04	0.02	0.04	0.1		

*Data in the upper section of the table represent means of n = 3 for each cell culture group. qPCR output expressed in* ΔΔ*Ct normalized to BSA control. Values within rows having different superscripts are significantly different by One-Way ANOVA and Tukey's mean separation test at α = 0.05. Values in the lower section of the table are the standard deviations of all means.*

BSA, bovine serum albumin; AA, arachidonic acid; EPA, eicosapentaenoic acid; DHA, docosahexaenoic acid; AEA, anandamide; 2AG, 2-arachidonoyl glycerol; CB1, cannabinoid receptor 1; CB2, cannabinoid receptor 2; NAPE-PLD, N-acyl phosphatidylethanolamine phospholipase D; FAAH, fatty acid amide hydrolase; DAGLα, diacylglycerol lipase-α; DAGLβ, diacylglycerol lipase-β; Akt1, RAC-alpha serine/threonine-protein kinase (protein kinase B); INS-R, insulin receptor; GLUT4, glucose transporter type 4; MyoD1, myogenic differentiation interleukin-6, IL-6; tumor necrosis factor-α, TNF-α.

#### Cannabinoid receptors CB1 and CB2

Compared to the BSA vehicle control cell cultures a higher expression of both CB1 and CB2 was observed with DHA, EPA, DHA, EPEA, and DHEA treatment of differentiated cells (Table 3). A lower expression of CB1 and CB2 was observed with AA, AEA, and 2-AG treatment of differentiated cell cultures. Treatment with AA, DHA, EPA, EPEA, and DHEA resulted in a higher amount of mRNA for CB1 and CB2 in proliferating myoblasts (Table S2). In these experiments n-3 PUFA and EPEA and DHEA resulted in the highest amount of cannabinoid receptor mRNA.

#### Synthesis and degradation enzymes

The treatment of differentiated C2C12 cell cultures with AA or AEA resulted in lower mRNA expression of NAPE-PLD while higher expression occurred with EPA treatment (Table 3). Expression of the synthesizing enzymes for 2-AG, DAGLα and DAGLβ, were also measured. DAGLα and DAGLβ were both observed to have higher expression with AA or EPA enrichment while 2-AG had an opposite effect and lowered mRNA expression of both enzymes. The enzyme responsible for degradation of AEA and 2-AG through hydrolysis is FAAH. This enzyme was found to be higher with AA, EPA, and AEA treatment of C2C12 cell cultures.

Similar to the differentiated cells, proliferating C2C12 cell cultures treated with AA or AEA, NAPE-PLD, which is the synthesizing enzyme for AEA, was lower compared to the no-fatty acid BSA vehicle control (Table S2). A higher expression of NAPE-PLD mRNA was observed with EPA and DHA treatment. AA, DHA, and AEA resulted in a higher expression of DAGLα, while AA, EPA, and AEA led to a higher DAGLβ expression. FAAH was observed here to be lower with AEA and AA treatment of proliferating C2C12 cell cultures.

#### ECS confirmation (MAPK + adenylyl cyclase)

In order to determine potential ECS receptor activity, several downstream events known to occur with activation were analyzed (Felder et al., 1993; Rolli-Derkinderen et al., 2003; Howlett, 2005). Here we see a lower expression of adenylyl cyclase and a higher expression of the MAPK enzymes, p42/p44, p38, and Jun N-terminal kinase (JNK) with only AEA and 2-AG in both proliferating and differentiated myoblasts (Tables 3, S2), which is consistent with the literature in confirming activation of the cannabinoid receptors (Felder et al., 1993; Howlett, 2005).

#### Glucose aspects

In proliferating C2C12 cultures, AA led to lower mRNA expression of insulin-R, GLUT1, and IRS-1. Contrastingly, DHA was found to have higher insulin-R, GLUT4, GLUT1, and IRS-1 mRNA expression. EPA was observed to have no significant changes to any of the genes involved in glucose uptake. AEA was observed to lower the mRNA expression of Akt-1, insulin-R, GLUT4, GLUT1, and IRS-1. 2-AG was also observed to have a negative effect on these glucose-related genes as Akt-1, GLUT1, and IRS-1 were all lower compared to the no-fatty acid vehicle control.

Many of the observations found in the proliferating cells were found to be the same in the differentiated C2C12 myoblasts. AA was found to lower the expression of Akt-1, insulin-R, GLUT1, and IRS-1. Again, DHA resulted in higher mRNA expression of Akt-1, insulin-R, GLUT4, GLUT1, and IRS-1. EPA led to higher Akt-1 expression. AEA resulted in lower Akt-1, insulin-R, GLUT4, GLUT1, and IRS-1 expression while 2-AG had a lowering effect on insulin-R, GLUT4, GLUT1, and IRS-1 mRNA.

#### Inflammation

In proliferating C2C12 myoblasts, inflammatory cytokine mRNA expression were analyzed and showed a higher IL-6 and TNF-α with AA, AEA, and 2-AG treatments while DHA enrichment led to a lower expression of the two inflammatory maker mRNA. In differentiated C2C12 myoblasts, IL-6 expression was shown to be lower by DHA enrichment and higher after AA, AEA, and 2-AG treatment. TNF-α expression was found to be lower with AA, DHA, AEA, and 2-AG treatment while EPA enrichment had no effect.

### PUFA and EC treatment resulted in varying CB1 and CB2 expression

**Figure 1:**
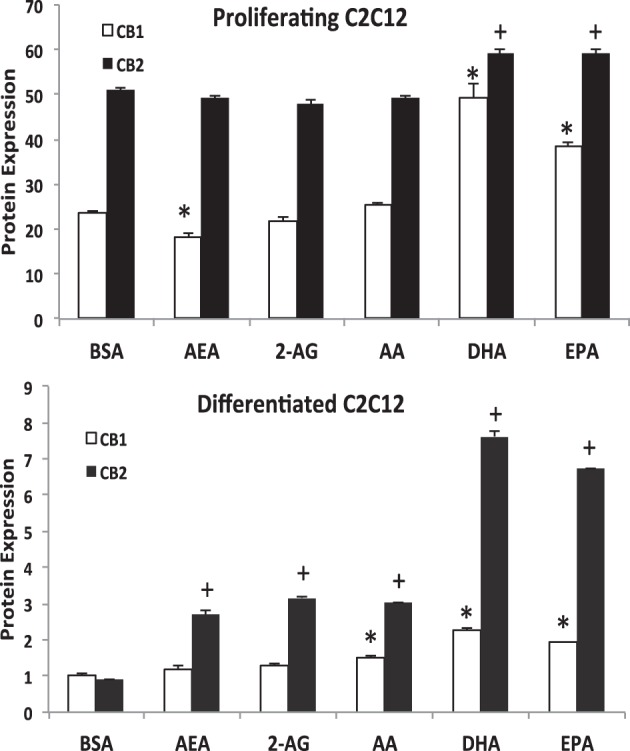
**Western blot analysis of CB1 and CB2 expression in proliferating and differentiated C2C12 myoblast cultures treated with PUFA and EC.** C2C12 cultures were treated with 25 μM of respective treatments for 24 h. The bars are the means ± *SD* (error bars) of 2 experiments performed in triplicate. The asterisk (^*^) signifies difference compared to the BSA control for CB1 while the plus sign (+) indicates difference compared to the BSA control for CB2. In proliferating cells compared to the BSA vehicle control, DHA and EPA values for both receptors were higher; CB1 was lower in the AEA treatment. In differentiating cells compared to the BSA vehicle control, all treatments showed higher CB2 protein expression but only AA, DHA and EPA treatments were higher for CB1.

Western blot analysis followed by densitometry was performed to measure CB1 and CB2 expression in differentiated and proliferating C2C12 myoblasts after being treated with either 25 μM of AA, EPA, DHA, AEA, 2-AG or a no-fatty acid vehicle control (Figure 1). In differentiated C2C12 myoblasts, AA, EPA, and DHA resulted in higher protein expression of CB1 compared to the BSA control group. CB2 protein expression was higher for AA, EPA, DHA, AEA, and 2-AG treated differentiated C2C12 myoblasts. Compared to the BSA vehicle control, EPA and DHA enrichment showed higher expression of CB1 and CB2 in proliferating C2C12 myoblasts while AEA treatment had lower expression.

**Figure 2:**
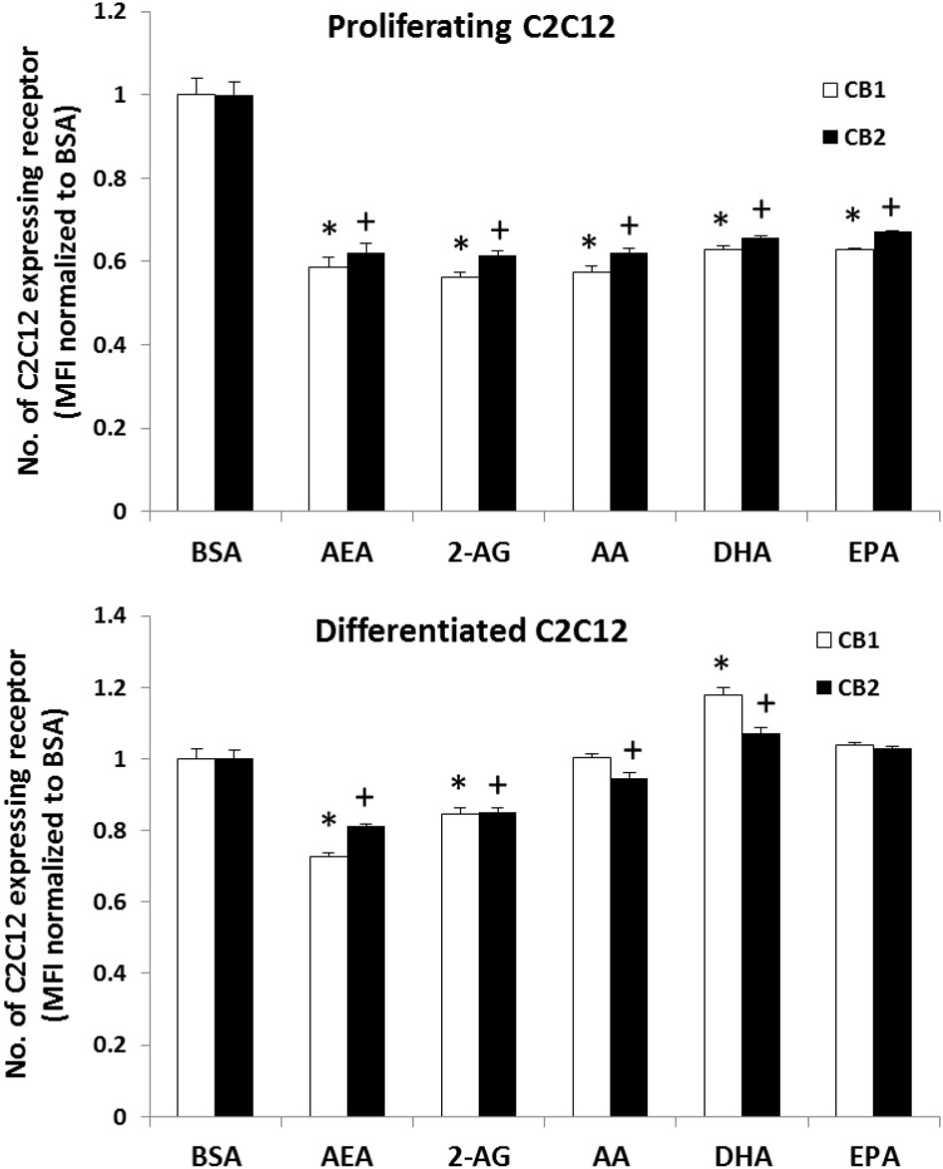
**FACS flow cytometry analysis of CB1 and CB2 expression in proliferating and differentiated C2C12 myoblast cultures treated with PUFA and EC.** C2C12 cultures were treated with 25 μM concentrations of respective treatments for 24 h. The bars are the means ± *SD* (error bars) of 3 experiments performed in triplicate. The asterisk (^*^) signifies difference compared to the BSA control for CB1 while the plus sign (+) indicates difference compared to the BSA control for CB2. In proliferating cells all treatments were different compared to the BSA vehicle control. In differentiating cells compared to the BSA vehicle control, DHA had higher CB1 and CB2 values and AEA and 2-AG were lower values but CB2 was lower in the AA cells.

FACS-flow cytometry was performed to measure CB1 and CB2 expression in differentiated and proliferating C2C12 myoblasts after being treated with either 25 μM of AA, EPA, DHA, AEA, 2-AG or a no-fatty acid vehicle control (Figure 2). A One-Way ANOVA revealed in differentiated C2C12 myoblasts, that DHA enrichment resulted in a higher expression of CB1 while AEA and 2-AG lower CB1 expression. CB2 expression was higher with DHA alone, while a lower amount was found with AA, AEA, and 2-AG treatment. A significant decrease in both CB1 and CB2 expression was observed with AA, EPA, DHA, AEA, or 2-AG treatment compared to the BSA control in proliferating cells.

### Glucose uptake is enhanced with DHEA treatment

**Figure 3:**
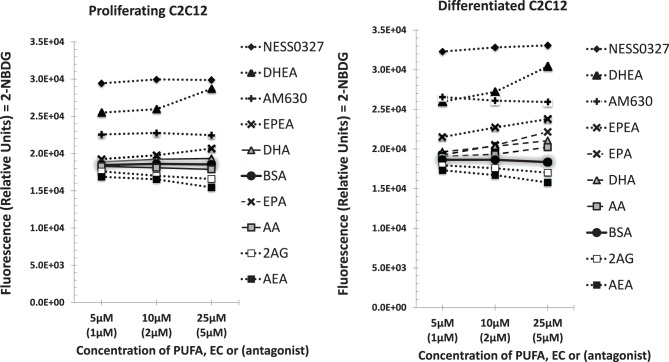
**Glucose uptake analysis of proliferating and differentiated C2C12 myoblast cultures treated with either three concentrations (5, 10, or 25 μM) of PUFA (AA, EPA, or DHA) or endocannabinoid (AEA, 2-AG, EPEA or DHEA) or with three concentrations (1, 2, or 5 μM) of cannabinoid receptor antagonist (AM630 or NESS0327) for 24 h.** Values shown are means of 3 experiments performed in triplicate. In proliferating cells compared to the BSA vehicle control **(gray shaded line)**, DHA, DHEA, EPEA, AM630, and NESS0327 had higher glucose uptake while AA, AEA, and 2-AG had lower glucose uptake. In differentiated cells compared to the BSA vehicle control, glucose uptake was lower for AEA and 2-AG treatments while all other treatments were higher. Data were analyzed by a One-Way ANOVA and Tukey's mean separation test at α = 0.05.The mean values and pooled s.e.m are shown in the supplemental materials Table S3.

Non-insulin stimulated glucose uptake was assessed in differentiated and proliferating C2C12 myoblasts after treatment with either 25 μM of AA, EPA, DHA, AEA, 2-AG, or 1 μM NESS0327 (CB1 inhibitor), 1 μM AM630 (CB2 inhibitor) or a BSA no-fatty acid vehicle control (Figure 3). In both differentiated and proliferating C2C12 cell cultures, AEA and 2-AG were found to result in lower glucose uptake compared to the BSA vehicle control cell cultures. However, EPEA, DHEA, NESS0327, and AM630 treatments showed higher glucose uptake. The results are presented as means ± *SD* in Table S3.

## Discussion

Enrichment of proliferating and differentiated C2C12 myoblasts with AA resulted in an increase in n-6 PUFA and the ratio of n-6/n-3 with a decrease in n-3 PUFA. Conversely, EPA or DHA treatment resulted in the opposite effect of the AA enrichment as an increase in n-3 PUFA with a decrease in n-6 PUFA was observed, resulting in a lower ratio of n-6/n-3 PUFA. These results are in accordance with previous findings in muscle and bone compartments of rodents fed n-6 or n-3 PUFA enriched diets (Watkins et al., 2000; Li et al., 2003; Watkins et al., 2006; Hutchins-Wiese et al., 2012). An aspect that differed from other fatty acid enrichment studies is the exclusion of FBS in enriching media. This exclusion allows for greater control of availing specific fatty acid exposure to cultures.

Our findings are the first to demonstrate that differentiated and proliferating C2C12 myoblasts cell cultures express key ECS-related components. Furthermore, the ECS-related components can be altered with PUFA or endocannabinoid exposure. An interesting finding was the observed increase in AA with 2-AG enrichment in both differentiated and proliferating cells. While this observation is the first to report this finding, it is very likely a result of 2-AG hydrolysis leading to the liberation and elevation of AA in cell lipids.

Several recent studies have demonstrated that endocannabinoid concentrations in tissues and in circulation are responsive to the types of dietary n-6 and n-3 PUFA when fed to animals or in cell cultures. Studies with mouse adipocytes incubated with AA or DHA (100 μM) for 72 h showed elevated levels of these PUFA in phospholipids (Matias et al., 2008). AA treatment in these cells led to an increase in 2-AG concentrations. Moreover, DHA treatment significantly decreased both AEA and 2-AG concentrations compared to the control. This study demonstrated that fatty acid enrichment of adipocytes in culture could also modify endocannabinoid concentrations with specificity. Several studies showed that dietary PUFA altered the levels of endocannabinoids in tissues including brain (Watanabe et al., 2003) adipose (Batetta et al., 2009), and liver (Artmann et al., 2008; Batetta et al., 2009). Aside from the ligands of the ECS, our laboratory (Hutchins et al., 2011) reported that EPA enrichment reduced the mRNA levels for the AEA synthesizing enzyme, NAPE-PLD, compared to AA and the vehicle control groups in MC3T3-E1 osteoblast-like cell cultures. While it isn't clear whether PUFA directly affect the cannabinoid receptors, CB2 mRNA was also reduced with EPA treatment. If and how long chain n-3 PUFA affect different cell types in a similar fashion, that is by impacting the ECS receptor expression, in culture or at different stages (differentiated or proliferating) and if similar responses occur *in vivo*, has not been investigated.

**Figure 4:**
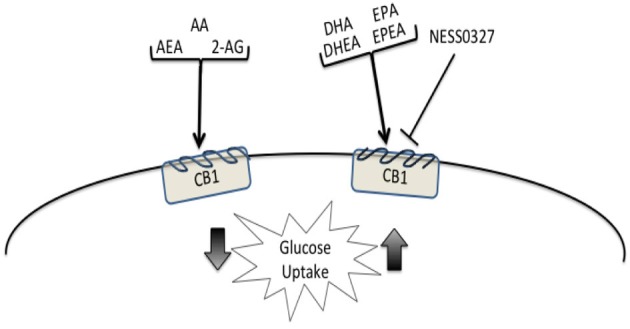
**The illustration summarizes the actions of PUFA, endocannabinoids, and cannabinoid antagonist and inverse agonist on glucose uptake and ECS gene expression in differentiated C2C12 myoblast cell cultures.** Differentiated C2C12 cultures treated with AA and its derived ligands, AEA and 2-AG, led to lower basal glucose uptake compared to the BSA vehicle control group. Treatment with DHA, DHEA, EPA, and EPEA resulted in higher basal glucose uptake compared to the BSA control group. The higher levels of glucose uptake were comparable to the uptake observed with the CB1 antagonist, NESS0327. The figure is developed from the key observations found by treatment of C2C12 myoblast cell cultures with PUFA, endocannabinoids, and cannabinoid receptor antagonism on CB1, inverse agonist on CB2, and the measurement of GLUT1, insulin-R mRNA, and glucose uptake in differentiated C2C12. In the table below upward arrows represent higher expression/activity compared to the BSA vehicle control while downward arrows signify lower expression/activity compared to the BSA control. Differentiated C2C12 cultures treated with AA and its derived ligands, AEA and 2-AG, led to lower CB1, GLUT1, and insulin-R mRNA expression as well as a reduction of basal glucose uptake compared to the BSA control group. Treatment with DHA, DHEA, EPA, and EPEA resulted in higher CB1, and DHA or DHEA higher GLUT1 and insulin-R mRNA expression as well as a greater level of glucose uptake compared to the BSA control group. NS indicates not significant from the BSA control group.
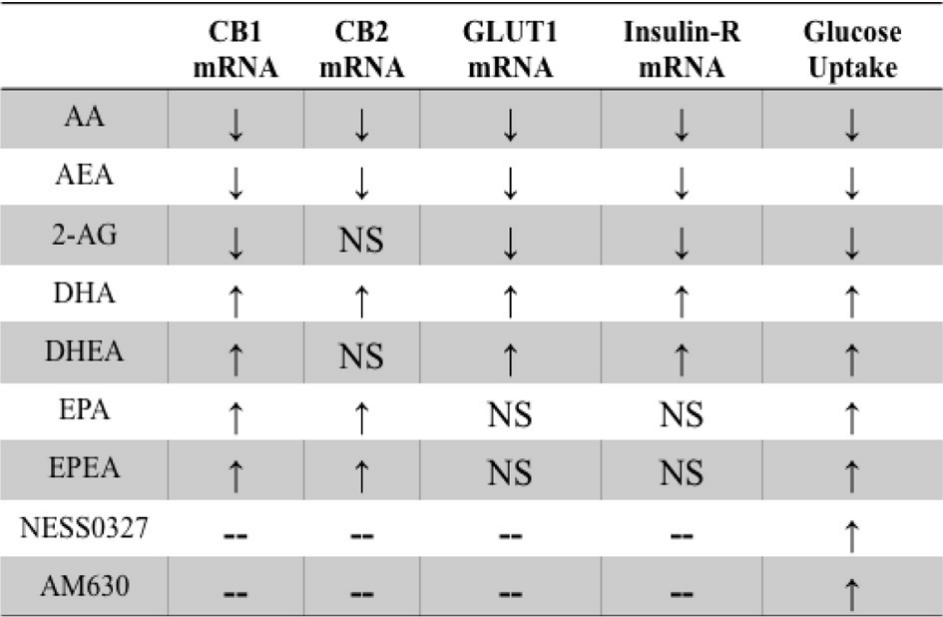

Our findings provide new evidence that the C2C12 myoblast cell line expresses ECS receptors and both synthesis and degradation enzymes. Further we report that treatment with PUFA or endocannabinoids were able to influence ECS tone and ultimately, signaling potential of the ECS in these cells. It is evident from the data presented here that with 24 h n-3 PUFA treatment, either EPA or DHA, the ECS tone is potentially upregulated. The mRNA and Western blot analysis for both CB1 and CB2 expression in proliferating and differentiated myoblast cultures were found to be higher than in the BSA vehicle control cell cultures. These findings are illustrated in Figure 4. The cannabinoid receptors were also found to be higher in differentiated cultures when analyzed by flow cytometry. Both EPA and DHA enrichment also resulted in higher endocannabinoid synthesis enzymes, NAPE-PLD and DAGLα/β, in proliferating while only EPA demonstrated the effect in differentiated C2C12 cell cultures.

Identification of components of the ECS in our cell system validates the model and was consistent with the literature, in which CB1 and CB2 was found in human skeletal muscle (Cavuoto et al., 2007; Eckardt et al., 2009) and differentiated L6 myotubes (Esposito et al., 2008). While the majority of ECS studies in muscle have focused on pharmacological interventions to monitor energy expenditure (Liu et al., 2005), mainly dealing with the short-lived antiobesity drug, Rimonabant (SR141716) (Esposito et al., 2008; Eckardt et al., 2009), the current investigation is the first to interrogate the ECS in muscle cells using dietary PUFA.

In proliferating C2C12 treated with AA, a decrease of NAPE-PLD was observed, the enzyme for AEA biosynthesis. It is possible that because of the abundant availability of the PUFA precursor, the synthesizing enzyme is being maximally saturated and less synthesis of AEA is required by the cell, resulting in a downregulation at the mRNA level of the enzyme. This is supported by the results with DHA and EPA treatments and both are potential precursors of endocannabinoids, DHEA and EPEA, respectively that were found to result in higher NAPE-PLD mRNA compared to the BSA control cells. The n-3 PUFA ethanolamides, docosahexaenoyl ethanolamide (DHEA) and eicosapentaenoyl ethanolamide (EPEA), have been previously shown to be weak ligands to cannabinoid receptors when compared to AEA (Sheskin et al., 1997), suggesting that the upregulation of ECS-related synthesis enzymes observed in this study are attributed to a compensatory response. Structure activity relationships suggest that the n-pentyl chain in AEA is necessary for optimal binding to the CB1 receptor (Felder et al., 1993); however, data relating to CB2 is inadequate. In addition, DHEA also binds to other receptors including PPARs (Artmann et al., 2008), highlighting the complexity of NAE signaling. Even with lower affinity to the cannabinoid receptors, perhaps the n-3 PUFA-derived ethanolamides are synthesized at a greater propensity leading to an inflated competition with other endocannabinoids resulting in the higher CB1 and CB2 expression seen in this study.

To confirm expression of activation of the ECS by AEA and 2-AG, the signaling pathway leading to the sequential activation of mitogen-activated protein kinase (MAPK) were also investigated here. Stimulation of either CB1 or CB2 has previously been shown to lead to the phosphorylation and activation of p42/p44 MAPK, p38 MAPK, and Jun N-terminal kinase (JNK) (Rolli-Derkinderen et al., 2003; Howlett, 2005; Eckardt et al., 2009). The endocannabinoids AEA, 2-AG, EPEA, and DHEA were all shown to increase the mRNA expression of these signaling pathways markers that regulate nuclear transcription factors. Additionally, a lowered expression of adenylyl cyclase mRNA with the endocannabinoid treatments was observed, concurring with observations of cannabinoid receptor activation (Felder et al., 1993; Howlett, 2005). Conversely, EPA and DHA showed a decrease in mRNA expression in p42/p44 MAPK, p38MAPK, and JNK in differentiated C2C12 with proliferating cells only being affected by DHA enrichment. Previously, it has been reported that fish oil decreased these MAPK-family signaling molecules (Lo et al., 2000). From the mRNA analysis, DHEA and EPEA had the highest fold change in p38 MAPK. p38 MAPK has been found to be lower in the liver of obese and type II diabetic mice (Lee et al., 2011). Activation of p38 MAPK was found to reduce endoplasmic reticulum stress and establish euglycemia in these severely obese mice. The MAPK/ERK signaling pathway has also been found to regulate insulin sensitivity into moderate glucose metabolism in *Drosophila* (Zhang et al., 2011). Further investigations of the ECS should focus on this pathway to ascertain the potential of restoring proper glucose homeostasis in obese and type II diabetics.

Signal transduction pathways involving MAPKs as well as the phosphatidylinositol 3-kinase (PI3-K) have been previously found to be key signaling cascades involved in the differentiation process of myoblasts (Zetser et al., 1999; Li and Johnson, 2006). However, the results of studies on the role of MAPKs in regulating skeletal muscle differentiation have been controversial. For example, activation of the ERK pathway seems to be important for mediating the repressive effect of growth factors on myogenesis. One study has suggested that ERK activation positively regulates myogenesis (Weyman and Wolfman, 1998). JNK has been involved in controlling diverse cellular functions, including cell proliferation, differentiation, and apoptosis (Garrington and Johnson, 1999). The activity of JNK has been reported to be activated during differentiation of C2 myoblasts and essential for survival (Bennett and Tonks, 1997; Khurana and Dey, 2004). Additionally, the role of MAPKs in the proliferation of skeletal muscle has not yet been fully studied, although inhibition of their activation is known to lead growth arrest in many cells, including myoblasts. Lipina et al. (2010) has also examined the effects of targeting CB1 and its downstream effects, finding that impeding CB1 activation enhanced both insulin-stimulated ERK1/2 and PI3-K/protein kinase-B activity in L6 myotubes. To add to these findings of insulin-sensitization in skeletal muscle cells, our study demonstrates that antagonizing CB1 resulted in increasing non-insulin stimulated glucose uptake.

The expression of the transcription factor myogenin is essential for the development of functional skeletal muscle as it is required for the proper fusion of myogenic precursor cells during myogenesis (Braun et al., 1989; Wright et al., 1989) Myogenin-deficient myoblasts, however, are unable to undergo efficient fusion to form functional muscle fibers *in vivo*. This result suggests that myogenin plays an essential role in the differentiation of myoblasts into myotubes (Hasty et al., 1993; Nabeshima et al., 1993). Similarly, MyoD1 is one of the earliest markers of myogenic commitment from the mesoderm and is expressed in activated satellite cells (Edmondson and Olson, 1989). Myogenin and MyoD1 have been shown to be expressed in regenerating skeletal muscle of mice (Fuchtbauer and Westphal, 1992). While there were no differences between treatments in the proliferating C2C12, DHA enrichment led to a higher expression of myogenin and MyoD1 in the differentiated cultures. Other findings in the literature suggest that DHA increases proliferation in C2C12 cells (Lee et al., 2009). However, the mechanism behind the ability of DHA to modulate skeletal muscle differentiation, whether it is via MAPK phosphorylation, has yet to be explained.

Some have reported that endocannabinoids are involved in the hypothalamic regulation of food intake and in peripheral lipogenesis in rodents (Di Marzo et al., 2001; Osei-Hyiaman et al., 2005). Thus the ECS has become a promising target for a pharmacological approach to obesity and diabetes. Antagonism of CB1 in rodents (Liu et al., 2005), primary myoblasts (Eckardt et al., 2009), and L6 myoblasts (Esposito et al., 2008) using Rimonabant (SR141716) have demonstrated effects on energy expenditure, targeting improved glucose metabolism, however, no information is available, thus far, in directing this endogenous machinery by dietary means, specifically with PUFA.

The key findings for glucose uptake in myoblasts are summarized in Figure 4. Enrichment of proliferating C2C12 myoblasts with EPA and DHA N-acylethanolamides, EPEA and DHEA, respectively, NESS0327, and AM630 resulted in higher basal glucose uptake in proliferating C2C12 cultures compared to the BSA control. In differentiated myoblasts, AA, EPA, DHA, EPEA, DHEA, NESS0327, and AM630 were all found to have higher basal glucose uptake compared to the control cultures (Figure 4). AEA and 2-AG both showed lower basal glucose uptake in both proliferating and differentiated C2C12 (Figure 4), which is consistent with the findings of elevated AEA and 2-AG in type II diabetic patients (Engeli et al., 2005; Bluher et al., 2006; Matias et al., 2006). Because of the elevated levels of endocannabinoids found in obese animals (Di Marzo et al., 2001) and diabetic humans (Engeli et al., 2005), activation of CB1 may be augmented resulting in an interference with glucose metabolism in muscle cells leading to a downregulation of glucose uptake. Several studies have demonstrated that skeletal muscle is responsive to ECS manipulation in mice (Liu et al., 2005). In a study in leptin deficient obese mice, treatment with the CB1 antagonist SR141716 induced a significant increase in glucose uptake in isolated soleus muscle after 7 days of treatment. In this study GLUT1 was thought to be a possible factor responsible for the response with SR141716 of increased glucose uptake in mouse muscle. In our study of C2C12 cultures the CB1 antagonist NESS0327 resulted in the greatest uptake of glucose, validating previous findings of the negative effects of CB1 on basal glucose uptake (Liu et al., 2005; Esposito et al., 2008; Eckardt et al., 2009). Higher expression of insulin-R, IRS-1, and GLUT4 with DHA treatment is in contrast to the lower glucose uptake with AEA and 2-AG. Our findings suggest that DHA and DHEA have a positive role in improving insulin-stimulated glucose uptake in myoblasts (Figure 4).

Additionally, AMPK activation has previously been found to improve glucose tolerance (Buhl et al., 2002). Long-term administration of 5-aminoimidazole-4-carboxamide ribonucleoside, a drug that activates AMPK, to insulin-resistant Zucker rats was shown to improve glucose tolerance and other reduce lipid accumulation. An increase in GLUT4 translocation in skeletal muscle is mainly responsible for the observed improved glucose tolerance (Holmes et al., 1999). Additionally, AMPK is activated during exercise at intensities >60% VO_2max_ in human subjects and rats (Fujii et al., 2000; Takekoshi et al., 2006). An interesting and relevant finding in our study was that both DHA and DHEA were the only treatments that resulted in higher GLUT4 mRNA levels. DHA has previously been shown to increase intestinal glucose absorption (Gabler et al., 2009). Most recently, DHA enrichment has been shown to interact with AMPK in C2C12 myoblasts to enhance uncoupling protein expression (Lee et al., 2013). DHEA was shown in the current investigation to improve basal glucose uptake. Whether DHEA is able to activate AMPK to effect GLUT1 remains to be determined. In addition, the observed increase in glucose-related mRNA suggests that DHEA may be a potential target in improving glucose clearance by muscle. Based on our findings future experiments must be conducted to investigate specific mechanisms on how PUFA affect signaling of the ECS in muscle and glucose utilization.

Based on the data presented herein, EPA and DHA enrichment of differentiated and proliferating C2C12 myoblasts led to a decrease in downstream markers of cannabinoid receptor activation. Conversely, endocannabinoids were all shown to increase downstream markers of cannabinoid receptor activation. While AEA and 2-AG treatment caused a marked decrease in adenylyl cyclase, indicating potential activation of the cannabinoid receptors, DHEA and EPEA resulted in a significantly higher mRNA expression, suggesting secondary messenger signaling to be moderated. Further, DHEA demonstrated an increase in basal glucose uptake at levels comparable to the CB1 antagonist, NESS0327 in myoblasts. Thus the current investigation demonstrates that long chain n-3 PUFA can mediate ECS gene expression and cellular activity in proliferating and differentiated myoblast cultures.

## Conflict of interest statement

The authors declare that the research was conducted in the absence of any commercial or financial relationships that could be construed as a potential conflict of interest.

## Supplementary material

Table S1 **Fatty acid composition of total lipids from proliferating C2C12 cell cultures**.

Table S2 **mRNA expression in proliferating C2C12 myoblast cultures treated with PUFA and EC**.

Table S3 **Glucose uptake assay in proliferating and differentiated C2C12 after FA or EC/inhibitor treatments (expressed as relative units of fluorescence)**.

## Abbreviations

2-AG: 2-arachidonoylglycerol
2-NBDG: 2-deoxy-2-[(7-nitro-2,1,3-benzoxadiazol-4-yl) amino]-D-glucose
AA: arachidonic acid
AEA: N-arachidonoylethanolamine; anandamide
ALA: α-linolenic acid
AMPK: AMP-activated protein kinase
BSA: bovine serum albumin
CB1: cannabinoid receptor 1
CB2: cannabinoid receptor 2
DAGLα: diacylglycerol lipase-α
DAGLβ: diacylglycerol lipase-β
DHA: docosahexaenoic acid
DHEA: docosahexaenoyl ethanolamide
DM: differentiation media
EC: endocannabinoid
ECS: endocannabinoid system
EPA: eicosapentaenoic acid
EPEA: eicosapentaenoyl ethanolamide
FAAH: fatty acid amide hydrolase
FACS: fluorescence activated cell sorting
FAME: fatty acid methyl ester
GM: growth media
IL: interleukin
IRS-1: insulin receptor substrate-1
LA: linoleic acid
MAPK: mitogen-activated protein kinase
NAPE-PLD: N-acyl phosphatidylethanolamine phospholipase
PUFA: polyunsaturated fatty acid
qPCR: quantitative polymerase chain reaction
TNF-α: tumor necrosis factor-α.
