# Supplementary material for: Docosahexaenoyl ethanolamide improves glucose uptake and alters endocannabinoid system gene expression in proliferating and differentiating C2C12 myoblasts
Source: Front Physiol. 2014 Mar 21;5:100. doi: 10.3389/fphys.2014.00100 (PMC3968752; doi:10.3389/fphys.2014.00100)
Supplement: Table S1 — Fatty acid composition of total lipids from proliferating C2C12 cell cultures. [file DataSheet1.PDF]

Supplemental

Table S1. Fatty acid composition of total lipids from proliferating C2C12 cell cultures

	Treatments							
FA	BSA	AA	EPA	DHA	AEA	2-AG	Pooled SEM	ANOVA p value
14:0	1.04^{ab}	0.77^{bc}	0.69^{c}	0.67^{c}	1.09^{a}	0.93^{ab}	0.06	0.0015
14:1n5								
15:0	0	0	0	0	0	0.05	0.02	0.46
16:0	14.22^{a}	11.96^{b}	11.49^{b}	11.78^{b}	13.66^{a}	13.80^{a}	0.15	<.0001
t16:1	2.31^{a}	1.40^{cd}	1.37^{d}	1.54^{c}	2.40^{a}	2.11^{b}	0.03	<.0001
16:1n7	4.97^{a}	2.32^{c}	2.36^{c}	2.44^{c}	5.09^{a}	4.10^{b}	0.08	<.0001
17:0	0.15^{b}	0.12^{b}	0.57^{a}	0.23^{b}	0.05^{b}	0.11^{b}	0.05	0.0001
18:0	13.52^{ab}	13.09^{bc}	13.74^{a}	13.86^{a}	12.75^{c}	13.33^{ab}	0.11	0.0001
18:1n9	26.64^{a}	14.68^{e}	15.88^{d}	16.97^{c}	26.11^{a}	23.83^{b}	0.15	<.0001
18:1n7	7.38^{a}	5.11^{c}	5.20^{c}	5.10^{c}	7.23^{a}	6.91^{b}	0.05	<.0001
18:2n6	1.16^{a}	0.60^{e}	0.72^{d}	0.89^{c}	1.16^{a}	1.02^{b}	0.01	<.0001
18:3n6								
18:3n3	0	0.06	0	0.	0	0	0.03	0.46
20:0								
20:1n9	0.57^{a}	0.23^{c}	0.34^{bc}	0.34^{bc}	0.53^{ab}	0.50^{ab}	0.05	0.0013
20:2n6								
20:3n6	0.76^{a}	0.50^{bc}	0.47^{c}	0.65^{bc}	0.74^{ab}	0.69^{ab}	0.02	<.0001
20:4n6	4.79^{c}	24.42^{a}	4.03^{d}	4.07^{d}	4.79^{c}	8.63^{b}	0.08	<.0001
20:3n3								
20:5n3	0.59^{c}	0.16^{d}	16.61^{a}	2.82^{b}	0.63^{c}	0.54^{c}	0.44	<.0001
22:0	0.22	0.38	0.17	0.26	0.54	0.48	0.09	0.095
22:1n9	0.28	0.28	0.22	0.26	0.47	0.35	0.06	0.15
22:4n6	0.44^{c}	6.24^{a}	0.54^{bc}	0.45^{c}	0.65^{b}	0.65^{b}	0.03	<.0001
22:5n6								
22:5n3	1.73^{b}	1.69^{b}	9.95^{a}	1.32^{c}	1.74^{b}	1.71^{b}	0.03	<.0001
22:6n3	2.35^{b}	1.97^{cd}	1.78^{d}	22.48^{a}	2.34^{bc}	2.23^{bc}	0.08	<.0001
24:0	0.35^{a}	0.15^{b}	0.29^{ab}	0.26^{ab}	0.36^{a}	0.39^{a}	0.03	0.0019
24:1n9	0.40^{ab}	0.29^{c}	0.34^{bc}	0.36^{abc}	0.40^{ab}	0.44^{a}	0.02	0.0014
TOTS	29.50^{a}	26.46^{c}	26.94^{c}	27.06^{c}	28.44^{b}	29.08^{ab}	0.22	<.0001
TOTM	40.24^{a}	22.91^{e}	24.34^{d}	25.47^{c}	39.82^{a}	36.13^{b}	0.21	<.0001
POLY	11.82^{e}	35.64^{a}	34.12^{b}	32.70^{c}	12.05^{e}	15.48^{d}	0.21	<.0001
TN6	7.15^{c}	31.76^{a}	5.77^{d}	6.07^{d}	7.34^{c}	10.99^{b}	0.13	<.0001
TN3	4.67^{c}	3.87^{d}	28.35^{a}	26.63^{b}	4.70^{c}	4.49^{c}	0.13	<.0001
n-6/n-3	1.53^{c}	8.21^{a}	0.20^{d}	0.23^{d}	1.56^{c}	2.45^{b}	0.10	<.0001

Data represent means of n = 3 for each group and the pooled SEM. Values within rows having different superscripts are significantly different by one-way ANOVA and Tukey's mean separation test at $\alpha = 0.05$. BSA, bovine serum albumin; AA, arachidonic acid; EPA, eicosapentaenoic acid; DHA, docosahexaenoic acid; AEA, anandamide; 2AG, 2-arachidonoyl glycerol

Table S2. mRNA expression in proliferating C2C12 myoblast cultures treated with PUFA and EC

Measurement	BSA	AA	DHA	EPA	AEA	2-AG	EPEA	DHEA	Pooled SEM	ANOVA p-value
CB1	1.0^{f}	1.4^{e}	3.2^{c}	2.8^{d}	1.2^{ef}	1.2^{ef}	3.6^{b}	5.4^{a}	0.06	<.0001
CB2	1.0^{f}	1.3^{e}	3.5^{b}	2.8^{c}	1.6^{de}	1.8^{d}	3.0^{c}	5.6^{a}	0.1	<.0001
NAPE-PLD	1.0^{b}	0.8^{bc}	2.6^{a}	2.4^{a}	0.5^{c}	0.8^{bc}	2.6^{a}	2.6^{a}	0.07	<.0001
FAAH	1.0^{de}	0.6^{f}	1.1^{cd}	1.1^{cd}	2.3^{a}	0.9^{e}	1.2^{bc}	1.4^{b}	0.04	<.0001
DAGL-α	1.0^{d}	2.0^{a}	1.3^{c}	0.9^{d}	1.5^{b}	0.7^{e}	0.9^{d}	1.0^{d}	0.03	<.0001
DAGL-β	1.0^{d}	1.5^{a}	1.2^{bcd}	1.3^{ab}	1.3^{abc}	1.1^{bcd}	1.1^{bcd}	1.1^{cd}	0.05	<.0001
Akt-1	1.0^{bc}	0.9^{cd}	1.0^{bcd}	1.0^{b}	0.7^{e}	0.9^{d}	1.0^{bcd}	2.1^{a}	0.02	<.0001
Insulin r	1.0^{cd}	0.8^{e}	1.5^{b}	1.1^{c}	0.6^{e}	0.9^{d}	1.0^{cd}	1.9^{a}	0.03	<.0001
IRS-1	1.0c	0.8d	1.4b	1.1c	0.3e	0.6d	1.0c	2.1a	0.03	<.0001
GLUT4	1.0c	1.0c	1.7b	0.9cd	0.7d	0.9cd	1.0c	2.5a	0.04	<.0001
GLUT1	1.0c	0.6d	1.3b	1.1c	0.1f	0.3e	1.0c	2.6a	0.03	<.0001
Myogenin	1.0	1.0	1.0	1.1	1.1	1.1	1.1	1.0	0.03	0.3
MyoD1	1.0	1.0	1.1	1.1	1.1	1.1	1.1	1.0	0.02	0.014
IL-6	1.0^{c}	1.7^{b}	0.6^{d}	1.1^{c}	2.1^{a}	1.9^{ab}	0.7^{d}	0.7^{d}	0.05	<.0001
TNF-α	1.0^{d}	1.3^{c}	0.7^{e}	1.1^{d}	2.0^{a}	1.8^{b}	1.1^{d}	0.8^{e}	0.03	<.0001
AMPK α2	1.0^{c}	1.0^{c}	1.7^{b}	0.9^{c}	0.6^{d}	0.7^{d}	0.9^{c}	1.9^{a}	0.03	<.0001
Adenylyl Cyclase	1.0^{b}	1.0^{b}	1.0^{ab}	1.1^{a}	0.2^{e}	0.2^{e}	0.8^{c}	0.6^{d}	0.02	<.0001
p42/p44 (MAPK)	1.0^{e}	1.3^{d}	0.7^{f}	0.9^{e}	1.7^{c}	1.7^{c}	2.0^{b}	2.9^{a}	0.03	<.0001
p38 (MAPK)	1.0^{e}	1.3^{d}	0.7^{f}	0.8^{ef}	1.6^{c}	1.6^{c}	2.0^{b}	3.6^{a}	0.04	<.0001
JNK (MAPK)	1.0^{e}	1.2^{d}	0.7^{f}	0.8^{ef}	1.7^{c}	1.6^{c}	2.0^{b}	3.4^{a}	0.04	<.0001
Standard deviation										
CB1	0.02	0.03	0.2	0.1	0.03	0.1	0.05	0.1		
CB2	0.1	0.1	0.1	0.2	0.03	0.1	0.1	0.04		
NAPE-PLD	0.02	0.1	0.3	0.1	0.03	0.01	0.2	0.1		
FAAH	0.1	0.04	0.03	0.1	0.1	0.1	0.01	0.01		
DAGL-α	0.1	0.1	0.1	0.1	0.03	0.1	0.03	0.04		
DAGL-β	0.1	0.1	0.1	0.1	0.04	0.1	0.02	0.02		
Akt-1	0.04	0.01	0.03	0.02	0.1	0.04	0.03	0.02		
Insulin r	0.1	0.03	0.1	0.04	0.03	0.01	0.02	0.05		
GLUT4	0.03	0.1	0.1	0.02	0.04	0.1	0.1	0.1		
Myogenin	0.1	0.1	0.1	0.03	0.04	0.01	0.03	0.05		
MyoD1	0.02	0.1	0.05	0.02	0.1	0.05	0.02	0.02		
GLUT1	0.1	0.1	0.05	0.03	0.003	0.01	0.05	0.1		
IRS-1	0.04	0.04	0.1	0.01	0.01	0.04	0.01	0.1		
IL-6	0.1	0.2	0.05	0.1	0.1	0.03	0.03	0.01		
TNF-α	0.01	0.1	0.03	0.05	0.1	0.1	0.03	0.02		
AMPK α2	0.03	0.1	0.1	0.02	0.03	0.05	0.1	0.1		
Adenylyl Cyclase	0.02	0.1	0.1	0.02	0.004	0.01	0.01	0.003		
p42/p44 (MAPK)	0.02	0.1	0.05	0.1	0.1	0.04	0.04	0.1		

p38 (MAPK)	0.1	0.1	0.03	0.02	0.1	0.04	0.04	0.1
JNK (MAPK)	0.04	0.1	0.04	0.03	0.1	0.02	0.1	0.1

Data in the upper section of the table represent means of n = 3 for each cell culture group. qPCR output expressed in ΔΔCt normalized to BSA control. Values within rows having different superscripts are significantly different by one-way ANOVA and Tukey's mean separation test at $\alpha = 0.05$. Values in the lower section of the table are the standard deviations of all means.

BSA, bovine serum albumin; AA, arachidonic acid; EPA, eicosapentaenoic acid; DHA, docosahexaenoic acid; AEA, anandamide; 2AG, 2-arachidonoyl glycerol; CB1, cannabinoid receptor 1; CB2, cannabinoid receptor 2; NAPE-PLD, N-acyl phosphatidylethanolamine phospholipase D; FAAH, fatty acid amide hydrolase; DAGLα, diacylglycerol lipase-α; DAGLβ, diacylglycerol lipase-β; Akt1, RAC-alpha serine/threonine-protein kinase (protein kinase B); INS-R, insulin receptor; GLUT4, glucose transporter type 4; MyoD1, myogenic differentiation interleukin-6, IL-6; tumor necrosis factor-α, TNF-α

Table S3. Glucose uptake assay in proliferating and differentiated C2C12 after FA or EC/inhibitor treatments (expressed as relative units of fluorescence)

	Treatments										Pooled SEM	ANOVA p value
	BSA	AA	EPA	DHA	AEA	2-AG	EPEA	DHEA	AM630	NESS0327		
Proliferating												
	16920 ±266 [f]	16196 ± 222 [g]	17126 ± 152 [f]	17798 ± 42 [e]	14176 ± 156 [i]	15568 ± 62 [h]	18650 ± 76 [d]	27925 ± 85 [b]	20934 ± 117 [c]	28907 ± 222 [a]	91	<.0001
Differentiated												
	17061 ± 247 [h]	18755 ± 132 [g]	20304 ± 44 [e]	19455 ± 270 [f]	14758 ± 143 [j]	15438 ± 115 [i]	21690 ± 200 [d]	29484 ± 261 [b]	25840 ± 191 [c]	32055 ± 165 [a]	109	<.0001

Glucose uptake analysis of proliferating and differentiated C2C12 myoblast cultures treated with either three concentrations (5, 10, or 25 μM) of PUFA or EC or with three concentrations (1, 2, or 5 μM) of cannabinoid receptor antagonist for 24 h. Values are the means ± SD of 3 experiments performed in triplicate. Values within rows having different superscripts are significantly different by one-way ANOVA and Tukey's mean separation test at $\alpha = 0.05$.
